# Levels of Urinary Metabolites of Organophosphate Flame Retardants, TDCIPP, and TPHP, in Pregnant Women in Shanghai

**DOI:** 10.1155/2016/9416054

**Published:** 2016-12-25

**Authors:** Liping Feng, Fengxiu Ouyang, Liangpo Liu, Xu Wang, Xia Wang, Yi-Ju Li, Amy Murtha, Heqing Shen, Junfeng Zhang, Jun Jim Zhang

**Affiliations:** ^1^Department of Obstetrics and Gynecology and Duke Global Health Institute, Duke University, Durham, NC, USA; ^2^MOE-Shanghai Key Laboratory of Children's Environmental Health, Xinhua Hospital, School of Medicine, Shanghai Jiao Tong University, Shanghai, China; ^3^Key Lab of Urban Environment and Health, Institute of Urban Environment, Chinese Academy of Sciences, Xiamen, China; ^4^Department of Biostatistics and Bioinformatics, Duke University, Durham, NC, USA; ^5^Nicholas School of the Environment and Duke Global Health Institute, Duke University, Durham, NC, USA; ^6^Duke Kunshan University, Kunshan, China

## Abstract

Flame retardants are widely used in consumer products to reduce their flammability. Previously used flame retardants have been sequentially banned due to their environmental and human toxicity. Currently, tris(1,3-dichloropropyl) phosphate (TDCIPP) and triphenyl phosphate (TPHP) are among the most commonly used flame retardants. TDCIPP and TPHP are reproductive toxins and have carcinogenic, neurotoxic, and endocrine-disrupting properties. Although high levels of TDCIPP and TPHP have been found in drinking water, seawater, and office air in China, data regarding human exposure are lacking. In this study, we assessed the level of urinary TPHP and TDCIPP metabolites (DPHP and BDCIPP, resp.) in a cohort of pregnant women (*N* = 23) from Shanghai, China, using liquid chromatography-tandem mass spectrometry. DPHP were detected in 100% urine samples, while only four urine samples had detectable level of BDCIPP in this cohort (17% detected). Geometric means of DPHP and BDCIPP concentrations were 1.1 ng/mL (interquartile range [IQR]: 0.6, 1.5 ng/mL) and 1.2 ng/mL (IQR: 0.6, 2.2 ng/mL), respectively. In this small cohort, urinary DPHP and BDCIPP levels were not significantly correlated with miscarriages, neonatal birthweight, gestational diabetes, or maternal age. These data suggest that exposure to TPHP is widespread, and they demonstrate the feasibility of using urinary biomarkers to measure exposures to modern flame-retardant chemicals.

## 1. Introduction

Flame retardants are widely used in consumer products such as textiles, plastics, furniture, electronics, cars, and construction materials to meet flammability standards since the 1960s [1]. Previously used flame retardants have been sequentially banned due to their environmental and human toxicity, including endocrine disruption, neurotoxicity, and carcinogenicity. Until recently, polybrominated diphenyl ethers (PBDEs) accounted for a large proportion of flame retardants used in household products [1, 2]. However, in the past several years, common PBDE mixtures have been banned or voluntarily phased-out in the United States and elsewhere [3]. Thus, the use of alternative flame retardants has been on the rise [2]. Organophosphate flame retardants (OPFRs) such as tris (1,3-dichloropropyl) phosphate (TDCIPP) and triphenyl phosphate (TPHP) are now among the most commonly used flame retardants [1, 4–6]. These flame retardants trends are similar in China. Based on China Flame Retardant Industry Report, 2012–2015, China has been a large producer and supplier of flame retardant in the world, with the output in 2011-2012 approximating 800,000–900,000 tons. Chlorinated and brominated flame retardants are still taking a lion's share in Chinese flame retardant market, with the proportion as high as 50% or so. Meanwhile, China fire retardant industry is upgrading product mix and technologies so as to follow the global development trend of halogen-free flame retardants. Strikingly, OPFRs have seen rapid development and grown into a hot variety, with major producers including Jiangsu Yoke Technology, Zhejiang Wansheng Co., Ltd, which are close to Shanghai. A recent study demonstrated the declined emission of PBDEs from industries in Southern China [7]. Consequently, ubiquitous environmental exposures of TDCIPP and TPHP have been reported in the United States [6, 8–17], Europe [18–22], and China [23–26].

TDCIPP and TPHP are persistent and bioaccumulative in the environment [27]. Toxicological data suggest that TDCIPP and TPHP are reproductive toxins [28, 29] and have carcinogenic [30], neurotoxic [31–33] and endocrine-disrupting properties [34–39]. Currently, available data are very limited on human exposure [10, 11, 40–42] and data on potential human health effects are lacking. To the best of our knowledge, data regarding human exposure to TPHP and TDCIPP in China have not been reported. Epidemiologic studies are critically needed. In our present pilot work, we utilized recently developed methods to extract and measure bis(1,3-dichloropropyl) phosphate (BDCIPP) and diphenyl phosphate (DPHP), the respective metabolites of TDCIPP and TPHP, in urine samples from a group of pregnant women in Shanghai.

## 2. Materials and Methods

### 2.1. Study Population

We recruited 23 pregnant women during November 2015 at the Xinhua Hospital, affiliated with Shanghai Jiao Tong University School of Medicine. Women provided demographic information and a spot urine sample during their second trimester clinical visit. Our final sample consisted of 23 urine specimens. All study protocols were approved by the institutional review board at the Xinhua Hospital, and all women provided informed consent.

### 2.2. Urinary TDCIPP and TPHP Metabolites Analysis

Urine samples were collected in sterile polyethylene specimen containers. Samples were packed on ice and transported to the lab in Xinhua Hospital, where they were aliquoted into 15 mL conical centrifuge tubes and frozen at −80°C until shipping. After all samples were collected, urine samples were packed on dry ice and transported to Institute of Urban Environment (IUE), Chinese Academy of Sciences in Xiamen, China for sample analysis. Modified extraction and measurement methods described previously [40, 41] were used. DPHP and d10-DPHP were purchased from TRC (Toronto, Canada). BDCIPP and d10-BDCIPP were purchased from Wellington Laboratories (Guelph, Ontario, Canada). Briefly, after thawing, 5 mL urine was buffered to pH = 6.5 with 1 M acetic acid if the samples were above pH 6.5. Urine samples were then spiked with 50 *µ*L mixture of working internal standard, d10-BDCPP and d10-DPP, solution (500 ng/mL). The internal standards were deconjugated using 5 *μ*L of *β*-glucuronidase/sulfatase from Helix pomatia (Type HP-2, aqueous solution, *β*-glucuronidase activity ≥ 100000 units/mL, sulfatase activity ≤ 7500 units/mL, Sigma-Aldrich, USA). The samples were incubated at 37°C for 90 min to deconjugate. A mixed-mode anion exchange solid phase extraction (Strata-X-AW, 60 mg/3 mL, Phenomenex Inc., Torrance, CA, USA) was preconditioned with dichloromethane (3 mL) and methanol (3 mL) sequentially. The treated urine was loaded onto the cartridge at a rate of 1 mL/min. After the loading of treated mixture, the SPE column was cleaned up with 3 mL distilled water. The analytes were eluted with methanol (3 mL) at 1 mL/min. The eluate was evaporated to dryness under a gentle stream of nitrogen at 45°C and then resuspended in 500 *µ*L methanol for the further analysis.

The target compounds were separated by an Accela UHPLC pumping system (Thermo Fisher Scientific, SanJosé, USA), coupled with an Accela Autosampler and Degasser. Separation of the compounds was carried out on a Hypersil Gold aQ C18 column (1.9 *µ*m, 100 mm × 2.1 mm, Thermo Fisher Scientific) which was kept at 30°C. The mobile phase of water and methanol was pumped at a flow rate of 0.3 mL/min. Optimized separation of BDCIPP and DPHP was obtained by using a linear gradient. The gradient was as follows: 0 min, 20% B; 0–6 min, 100% B (linear); hold for 2 min; 8–8.1 min, 20% B (linear); and hold for 9.9 min. The total run time for each injection was (18 min) and the injection volume was 20 *µ*L.

The target compounds were analyzed by a triple quadrupole mass analyzer (TSQ Vantage, Thermo Fisher Scientific) which was fitted with atmospheric pressure chemical ionization in negative ion mode. The following working conditions were applied: spray voltage at 2.5 (−) kV; vaporizer and capillary temperature at 300 and 325°C, respectively; sheath and auxiliary gas at 45 and 15 arbitrary units (a.u.), respectively; cycle time of 1.0 second. Argon pressure in the collision cell (Q2) was set at 1.5 mTorr and the mass resolution at the first (Q1) and third (Q3) quadrupole was set at 0.70 Da at full width at 50% of maximum (FWHM). Precursor ion, S-lens RF amplitude, and collision energy (CE) in Q2 were optimized individually per compound and/or transition (Table 1). Quantification and confirmation data were acquired in selected reaction monitoring (SRM) mode, and the transitions are displayed as Table 1. Instrument control and data processing were carried out by means of Xcalibur Software 2.2 SP 1.48 (Thermo Electron, San José, USA).

For quality assurance purposes we evaluated the recovery of d10-BDCIPP and d10-DPHP in spiked pooled samples and measured the amount of BDCIPP and DPHP levels in laboratory blanks (*n* = 3). The laboratory blank was deionized water obtained from a Millipore water purification system (ELGA LabWater, Lane End, HP14 3JH, UK). The deionized water was further cleaned up by SPE as real sample extraction before being used to prepare the buffer solutions and other aqueous solutions. The average recoveries of d10-BDCIPP and d10-DPHP were 112.5 ± 0.6 and 81.1 ± 6.6%, respectively. Very small amounts of DPHP were detected in laboratory blanks (0.012 ng/mL), while BDCIPP was detected in relatively higher levels in laboratory blanks (0.13 ng/mL). The method detection limit (MDL) or limit of detection (LOD) was calculated using three times the standard deviation of the blanks, which was 0.057 ng/mL for DPHP and 0.11 ng/mL for BDCIPP. Levels of BDCIPP and DPHP in urine were corrected for recovery of the mass labeled internal standards. All concentration data were corrected with laboratory blanks. If subtracting the blank value resulted in a negative value, the concentration of the sample was regarded as below MDL. We have chosen to present results using the MDL/$\sqrt{2}$ method for values below the MDL [40]. Sample specific gravity (SG) was measured in each urine sample prior to analysis using a digital handheld refractometer (Atago Co., Ltd., Tokyo, Japan). All data analyses were conducted using specific gravity adjusted concentrations account for urine dilution [40].

### 2.3. Statistical Analyses

We calculated descriptive statistics for each metabolite, including the geometric mean concentration and selected percentiles (10th, 25th, 50th, 75th, and 90th percentiles). We also examined the distribution of urinary BDCIPP and DPHP graphically. Due to the small sample size and the nonnormality of BDCIPP and DPHP, the exact Wilcoxon rank-sum test was used to compare the median differences of urinary BDCIPP or DPHP concentrations between 3 miscarriages and 15 term pregnancies. The same test was also used to compare the median differences of urinary BDCIPP or DPHP concentrations between subjects with/without gestational diabetes. Multivariable logistic regression modeling was performed to test the effect of DPHP on gestational diabetes with adjustment of neonatal birthweight. The pairwise Spearman correlation for urinary BDCIPP or DPHP with maternal age and neonatal birthweight were computed and tested. The correlation of BDCIPP and DPHP levels among subjects was also tested.

The level of significance was a two-sided *p* value < 0.05. All analyses were performed using the SAS 9.4 software (SAS Institute, Inc., Cary, NC, USA).

## 3. Results

Twenty-three women participating in our study ranged in age from 25 to 40 years at conception and all had a college education and above. Prepregnancy body mass index of all subjects is in the range of 18–25. Subjects' occupation and life style information was not detailed enough to indicate sources of exposure. Three subjects had elective abortions. Five subjects could not be followed up for birth outcomes. One subject delivered a macrosomia infant. All other subjects (*n* = 14) delivered healthy, full-term infants. However, among the 15 subjects, 4 had gestational diabetes and 1 had preeclampsia during pregnancy.

**Figure 1:**
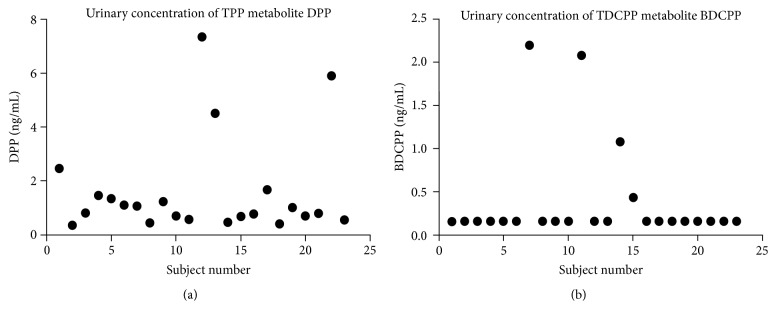
Distribution of urinary concentration of TPHP and TDCIPP metabolites DPHP and BDCIPP, respectively.

DPHP was detected in all urine samples (100% detected); four urine samples had detectable level of BDCIPP (17% detected). A lower bound of 0.16 was used for those subjects with nondetectable urinary BDCIPP based on the MDL. The low detect rate of urinary BDCIPP in this cohort could be caused by the higher detection limit of BDCIPP and matrix effect in urine sample in our study. Urinary concentrations of these compounds varied from individual to individual in detectable samples. The distribution of urinary BDCIPP and DPHP concentrations is shown in Table 2 and Figure 1. Levels of BDCIPP and DPHP were not correlated in this cohort (*r* = −0.2, *p* = 0.4). The geomean of DPHP or BDCIPP is higher in patients who had miscarriages than subjects who delivered at full term (mean [SD]: 1.3 [1.4] versus 2.0 [2.2] for DPHP; 0.4 [0.6] versus 0.8 [1.1] for BDCIPP). However, the median of these compounds is similar between these two groups (0.8 versus 0.7 for DPHP and 0.2 versus 0.2 for BDCIPP). And no comparisons are statistically significant (*p* > 0.5). The mean and median of DPHP are higher in patients with gestational diabetes than those without gestational diabetes (mean (SD): 2.6 (2.4) versus 1.4 (1.7) and median: 1.8 versus 0.8). However, median differences of urinary BDCIPP or DPHP concentrations between pregnancy complicated with/without gestational diabetes were not significantly different (*p* = 0.6 for BDCIPP and *p* = 0.2 for DPHP). Since birthweight for DPHP is close to nominal significance for gestational diabetes, we also performed multivariable logistic regression modeling to test the effect of DPHP on gestational diabetes with adjustment of birthweight; it remained nonsignificant (odds ratio [OR] 4.6, 95% confidence interval [CI]: 0.16–133.8, *p* = 0.4). No significant correlation was observed between urinary BDCIPP or DPHP with maternal age (*r* = 0.2, *p* = 0.4 for BDCIPP; *r* = −0.16, *p* = 0.5 for DPHP) and neonatal birthweight (*r* = −0.3, *p* = 0.2 for BDCIPP; *r* = 0.2, *p* = 0.45 for DPHP). Detailed statistical analysis data are presented in the Supplementary Data (in Supplementary Material available online at http://dx.doi.org/10.1155/2016/9416054).

## 4. Discussion

DPHP was detectable in all urine samples collected, suggesting possible widespread exposure to DPHP itself or its parent compounds including TPHP in this study region, while exposures to TDCIPP might be selective, with only a 17% detectable rate in pregnant women in Shanghai. These results for TPHP are consistent with several small studies from the United States that also reported relatively variable but near ubiquitous exposure in nonpregnant populations [10, 11, 41]. However, the geometric mean concentrations were higher in this Shanghai pregnant cohort than in nonpregnant subjects. The results in this Shanghai pregnant cohort are similar to those recently reported in pregnant women in the United States (US) [40] and Canada [42]. In the Canadian cohort of pregnant women, the detection frequency of BDCIPP (29.2%) is also significantly less than DPHP (91.7%), which is similar to our observation. The geomean concentration of DPHP urinary levels (2.72 ng/mL) observed in Canadian pregnant cohort is higher than the US (1.9 ng/mL) and our Shanghai pregnant cohort (1.1 ng/mL). In contrast, the geomean concentration of BDCIPP urinary levels (0.27 ng/mL) observed in Canadian pregnant cohort is lower than the US (1.1 ng/mL) and our Shanghai pregnant cohort (1.2 ng/mL) [40, 42]. The authors of the US study suggest that differences in excretion rates and kidney function during pregnancy may explain the higher metabolite levels observed in pregnant women relative to nonpregnant cohorts [40]. However, the relatively similar geomean of BDCIPP levels between the Canadian pregnant cohort and the other nonpregnant cohorts indicates that the measurements among pregnant women may be more reflective of actual exposures rather than confounding factors of altered excretion associated with pregnancy stage [42].

We observed nonstatistically significant correlations between urinary DPHP and BDCIPP concentrations, which may be explained by the higher detection limit of BDCIPP and matrix effect in urine sample in our study. This observation may also be explained by differences in the sources of exposure to TDCIPP and TPHP. All subjects in our study cohort were neither living near fire retardant industries nor factory workers, so we suspect the source of exposure in this cohort are unlikely to be direct industrial plant exposures. Data relating to OPFRs in China remain scarce [25]. Few studies that reported fairly ubiquitous environmental exposures of TDCIPP and TPHP in drinking water [23], seawater [25], and office air [24] do not explain the selective exposure of TDCIPP in this study cohort. While both TDCIPP and TPHP are used as additive flame retardants in household products, TPHP is also used as a plasticizer and lubricant and in hydraulic fluids [1], which may contribute to the more ubiquitous human exposures in this cohort. Differences in the metabolism and excretion of TDCIPP and TPHP also provide possible explanations for the nonsignificant correlation observed in urinary biomarkers of exposure [40]. Interestingly, two recent studies demonstrated a route of exposure of TDCIPP and TPHP through diet and food consumptions in China [43, 44]. The differences in the levels of TDCIPP and TPHP in foods and dietary differences among subjects might in part contribute to the observed discrepancy in urinary DPHP and BDCIPP concentrations in our study.

Although the toxicokinetics of TDCIPP and TPHP in the human body have yet to be explored, data suggest that they are rapidly metabolized to (BDCIPP and DPHP) and are rapidly eliminated from the body [41, 45, 46]. Nonetheless, a previous study observed moderate to strong reliability in the levels of BDCIPP and DPHP in urine samples collected throughout pregnancy and shortly after giving birth [40]. Therefore, the study authors concluded that a single measure of levels during pregnancy may be sufficient in characterizing levels throughout pregnancy [40]. These data suggest that TDCIPP and TPHP may come from more continuous sources of exposure than diet, such as contaminated dust in the home or workplace environments. This would seem a reasonable hypothesis given that human exposure to PBDE flame retardants in the United States has been demonstrated to occur primarily from exposure to household dust, both in adults and in children [5, 47].

Adjustments for urine dilution have been recommended in the assessment of xenobiotics and are commonly included in epidemiologic investigations [48]. In this study, we chose to adjust with urinary specific gravity, which is thought to be less impacted by changes with age, body composition, physical activity, urine flow, time of day, diet, disease, and pregnancy than other measures such as creatinine [48, 49]. However, as a previous study suggested, other measures of urine dilution and methods of correction should be included in future studies of BDCIPP and DPHP. This study is also limited by the small sample size (23 women with a total of 23 measurements) and composition—specifically highly-educated and normal-weight pregnant women—which may differ substantially from other populations. Sample size may be one of the explanations of the observed nonstatically significant association of levels of urinary TDCIPP and TDD with subject characteristics and pregnancy outcomes. We observed a trend of negative association of TDD exposure with subject age. However, it did not reach statistical significance. The other limitation of this study is that the subjects' occupation and life style information was not detailed enough to indicate sources of exposure. Follow-up of our findings in larger cohorts with longitudinal data will provide additional insights.

## 5. Conclusions

Our results demonstrate ubiquitous exposure to TPHP (100% detection of TPHP metabolite DPHP) and exposure to TDCIPP (17% above-detection limit of the urinary metabolite BDCIPP) in pregnant women is likely in Shanghai, China. Additionally, we observed a modest degree of variability in urinary metabolites of BDCIPP and DPHP among women in our study population. The association of urinary levels of BDCIPP and DPHP with pregnancy outcomes and subject characteristics was not observed, as our analyses were limited by our small sample size and relatively homogeneous study population of Shanghai pregnant women. This is the first feasibility study showing that exposure to modern flame-retardant chemicals can be detected in pregnant women living in Shanghai. Further studies can use these urinary biomarkers of exposure to help identify sources of exposure to OPFRs and/or assess OPFRs' impact on health outcomes in a larger cohort.

## Supplementary Material

Statistical analyses were performed to examine the association between urinary levels of DPHP or BDCIPP and miscarriage or gestational diabetes. No comparisons are statistically significant.

## Figures and Tables

**Table 1:** Parameters for MRM acquisition of the analysis.

Analyte	Detection mode	Precursor ion (*m/z*)	Slens (Hz)	SRM1^*∗*^ (*m/z*)	Collision energy (eV)	SRM2 (*m/z*)	Collision energy (eV)
BDCIPP	—	319	59	35	55	37	14
D_10_-BDCIPP	—	329	57	35	63	/	/
DPHP	—	249	79	93	37	155	25
D_10_-DPHP	—	259	79	98	40	/	/

^*∗*^Quantitative ions.

**Table 2:** Distribution of urinary DPHP and BDCIPP concentrations (ng/mL) among 23 urine samples from 23 pregnant women in Shanghai.

Urinary metabolite	Percent detect^*∗*^	Geometric mean^#^	Percentiles					Maximum
			10th	25th	50th	75th	95th	
DPHP	100	1.1	0.45	0.59	0.83	1.48	5.92	7.3
BDCIPP	17	1.2	0.43	0.59	1.58	2.17	2.20	2.2

^*∗*^Percentage based on 23 urine samples analyzed for DPHP and BDCIPP.

^#^Geometric mean was calculated based on detectable concentrations.

## Abbreviations

BDCIPP: Bis(1,3-dichloropropyl) phosphate
CI: Confidence interval
DPHP: Diphenyl phosphate
IQR: Interquartile range
MDL: Method detection limit
OPFRs: Organophosphate flame retardants
PBDEs: Polybrominated diphenyl ethers
SG: Specific gravity
TDCIPP: Tris(1,3-dichloropropyl) phosphate
TPHP: Triphenyl phosphate.
