# Supplementary material for: Levels of Urinary Metabolites of Organophosphate Flame Retardants, TDCIPP, and TPHP, in Pregnant Women in Shanghai
Source: J Environ Public Health. 2016 Dec 25;2016:9416054. doi: 10.1155/2016/9416054 (PMC5220514; doi:10.1155/2016/9416054)
Supplement: Supplementary file 1 — Statistical analyses were performed to examine the association between urinary levels of DPHP or BDCIPP and miscarriage or gestational diabetes. No comparisons are statistically significant. [file 9416054.f1.pdf]

Due to the small sample size and the non-normality of ~~DPP~~DPHP and ~~BDCPP~~BDCIPP, exact Wilcoxon rank sum test was used to compare the median difference of each variable listed in Tables 1 and 2. While no significant results were found for ~~DPP~~DPHP and ~~BDCPP~~BDCIPP, the mean of ~~DPP~~DPHP and ~~BDCPP~~BDCIPP are higher in patients who had miscarriages than those who delivered at full term (mean (SD): 1.3 (1.4) vs. 2.0 (2.2) for ~~DPP~~DPHP; 0.4(0.6) vs. 0.8(1.1) for ~~BDCPP~~BDCIPP). The median of these compounds is similar between these two groups (0.8 vs. 0.7 for ~~DPP~~DPHP and 0.2 vs. 0.2 for ~~BDCPP~~BDCIPP).

Table 1: comparison between miscarriage and normal deliver groups

	0<br>(N=15)	1<br>(N=3)	Total<br>(N=18)	p value
**age**				0.2868
N	15	2	17	
Mean (SD)	29.7 (4.0)	26.5 (2.1)	29.3 (3.9)	
Median	29.0	26.5	28.0	
Q1, Q3	26.0, 32.0	25.0, 28.0	26.0, 32.0	
Range	(25.0-40.0)	(25.0-28.0)	(25.0-40.0)	
**~~DPP~~DPHP**				0.9118
N	15	3	18	
Mean (SD)	1.3 (1.4)	2.0 (2.2)	1.4 (1.5)	
Median	0.8	0.7	0.8	
Q1, Q3	0.6, 1.4	0.6, 4.5	0.6, 1.4	
Range	(0.4-5.9)	(0.6-4.5)	(0.4-5.9)	
**~~BDCPP~~BDCIPP**				0.7770
N	15	3	18	
Mean (SD)	0.4 (0.6)	0.8 (1.1)	0.4 (0.7)	
Median	0.2	0.2	0.2	
Q1, Q3	0.2, 0.2	0.2, 2.1	0.2, 0.2	
Range	(0.2-2.2)	(0.2-2.1)	(0.2-2.2)	

(report generated on 28MAY2016)

Due to the small sample size and the non-normality of ~~DPP~~DPHP and ~~BDCPP~~BDCIPP, exact Wilcoxon rank sum test was used to compare the median difference of each variable listed in Tables 1 and 2. While no significant results were found for ~~DPP~~DPHP and ~~BDCPP~~BDCIPP, the mean and median of ~~DPP~~DPHP are higher in patients with gestational diabetes than those without gestational diabetes (mean (SD): 2.6 (2.4) vs. 1.4 (1.7) and median: 1.8 vs. 0.8).

Table 2: comparison between patients with and without gestational diabetes

	0 (N=19)	1 (N=4)	Total (N=23)	p value
**age**				0.7091
N	17	4	21	
Mean (SD)	29.1 (3.8)	31.3 (6.8)	29.5 (4.4)	
Median	28.0	30.0	28.0	
Q1, Q3	26.0, 31.0	26.0, 36.5	26.0, 32.0	
Range	(25.0-40.0)	(25.0-40.0)	(25.0-40.0)	
**Bweight**				0.0557
N	11	4	15	
Mean (SD)	3259.5 (431.4)	3872.5 (631.7)	3423.0 (545.1)	
Median	3095.0	3680.0	3300.0	
Q1, Q3	2995.0, 3530.0	3412.5, 4332.5	3060.0, 3560.0	
Range	(2865.0-4360.0)	(3380.0-4750.0)	(2865.0-4750.0)	
**~~DPP~~DPHP**				0.1884
N	19	4	23	
Mean (SD)	1.4 (1.7)	2.6 (2.4)	1.6 (1.8)	
Median	0.8	1.8	0.8	
Q1, Q3	0.6, 1.4	0.9, 4.2	0.6, 1.5	
Range	(0.4-7.3)	(0.7-5.9)	(0.4-7.3)	
**~~BDCPP~~BDCIPP**				0.5623
N	19	4	23	
Mean (SD)	0.4 (0.6)	0.2 (0.0)	0.4 (0.6)	
Median	0.2	0.2	0.2	
Q1, Q3	0.2, 0.2	0.2, 0.2	0.2, 0.2	
Range	(0.2-2.2)	(0.2-0.2)	(0.2-2.2)	

Multivariable logistic regression:

Since birthweight for ~~DPP~~DPHP is close to nominal significance for gestational diabetes, we conducted multivariable logistic regression model to test the effect of ~~DPP~~DPHP on gestational diabetes with adjustment of birthweight.

Table 3: Multivariable logistic regression for gestational diabetes

Variable	OR (95% CI)	p
Birth weight	1.00 (0.99, 1.006)	0.158
~~DPP~~DPHP	4.63 (0.16, 133.88)	0.3725

Table 4: Pairwise Spearman correlation for age, birthweight, ~~DPP~~DPHP, and ~~BDCPP~~BDCIPP

Correlation coefficient (r)<br>(p value)	Age	Birthweight	~~DPP~~DPHP	~~BDCPP~~BDCIPP
Age	1	0.161 (0.565)	-0.160 (0.489)	0.197 (0.391)
Birthweight		1	0.211 (0.451)	-0.319 (0.246)
~~DPP~~DPHP			1	-0.275 (0.204)
~~BDCPP~~BDCIPP				1
